# NALIRIFOX versus FOLFIRINOX: reconstructed individual patient data meta-analysis of efficacy and safety in first-line treatment of unresectable or metastatic pancreatic ductal adenocarcinoma

**DOI:** 10.3389/fonc.2026.1902839

**Published:** 2026-09-18

**Authors:** Abdullah Esmail, Jian Guan, Zaid Alabed, Yazan Hamdaneh, Ebtesam Al-Najjar, Saifudeen Abdelrahim, Nour Mustafa, Bayan Khasawneh, Maen Abdelrahim

**Affiliations:** 1 Section of GI Oncology, Department of Medicine, Houston Methodist Cancer Center, Houston, TX, United States; 2 Department of Medicine, Weill Cornell Medical College, New York, NY, United States; 3 Faculty of Medicine, The University of Jordan, Amman, Jordan

**Keywords:** FOLFIRINOX, meta-analysis, NALIRIFOX, pancreatic ductal adenocarcinoma, reconstructed individual patient data

## Abstract

**Background:**

Pancreatic ductal adenocarcinoma (PDAC) remains one of the most lethal human malignancies, with a 5-year overall survival rate below 10%. NALIRIFOX and FOLFIRINOX, both in full-dose and modified (mFOLFIRINOX) formulations, are guideline-endorsed Category 1 first-line regimens for fit patients with metastatic PDAC. However, no direct head-to-head randomized trial has compared these regimens. This systematic review and reconstructed individual patient data meta-analysis represents the first comprehensive simultaneous comparison of NALIRIFOX and FOLFIRINOX/mFOLFIRINOX across efficacy and safety outcomes.

**Methods:**

A systematic search was conducted across PubMed, Embase, Scopus, ClinicalTrials.gov, and ASCO/ESMO proceedings from January 2011 to May 2025. Eligible studies enrolled treatment-naïve adults with unresectable or metastatic PDAC receiving first-line NALIRIFOX or FOLFIRINOX. Individual patient-level time-to-event data for overall survival (OS) and progression-free survival (PFS) were reconstructed from published Kaplan-Meier curves and pooled across studies. Treatment effects were estimated using mixed-effects Cox regression models accounting for between-study variability. Grade ≥3 adverse events were pooled using a random-effects model.

**Results:**

Nine studies encompassing 2,733 patients were included in the primary OS analysis. Of these, 1,138 (42%) received FOLFIRINOX, 1,180 (43%) received mFOLFIRINOX, and 415 (15%) received NALIRIFOX. Six studies (n = 1,028 patients) reported PFS and were included in that analysis. The overall median OS (mOS) across all groups was 12.38 months (95% CI: 12.06–12.85). By regimen: mOS was 10.98 months (95% CI: 10.43–11.53) for FOLFIRINOX, 14.09 months (95% CI: 13.52–15.02) for mFOLFIRINOX, and 11.81 months (95% CI: 10.42–12.52) for NALIRIFOX. When FOLFIRINOX and mFOLFIRINOX were pooled, the combined group achieved a mOS of 12.59 months (95% CI: 12.21–13.27), which showed no statistically significant difference compared with NALIRIFOX (HR 1.13, 95% CI: 0.71 –1.81; p = 0.6). Moreover, no statistically significant difference was found between NALIRIFOX and full-dose FOLFIRINOX alone (HR = 1.09, 95% CI: 0.66 –1.79; p = 0.73). The overall mPFS was 7.66 months (95% CI: 7.30–8.21). By regimen: mPFS was 8.24 months (95% CI: 7.55–9.35) for FOLFIRINOX, 6.64 months (95% CI: 5.78–7.74) for mFOLFIRINOX, and 7.49 months (95% CI: 7.13–8.78) for NALIRIFOX; the pooled FOLFIRINOX/mFOLFIRINOX group achieved a mPFS of 7.71 months (95% CI: 7.23–8.41). Safety analysis confirmed distinct toxicity profiles: FOLFIRINOX carried a higher burden of grade 3/4 hematologic toxicity (neutropenia up to 45–53%; thrombocytopenia 11.8%), while NALIRIFOX was associated with lower hematologic toxicity but substantially higher grade 3/4 diarrhea (~20.3%) and hypokalemia (15.1%).

**Conclusion:**

No statistically significant differences in OS or PFS were observed between NALIRIFOX and FOLFIRINOX. The numerically higher OS observed in the pooled FOLFIRINOX/mFOLFIRINOX group was largely driven by the mFOLFIRINOX subgroup. NALIRIFOX’s lower hematologic toxicity may reduce downstream resource utilization. Prospective head-to-head trials and biomarker-driven studies are needed.

**Systematic review registration:**

[URL], identifier (identifier number)

## 1 Introduction

Pancreatic cancer remains one of the most lethal malignancies, with almost 80% of cases diagnosed at either unresectable locally advanced or metastatic stages and a 5-year survival rate of less than 10%. Pancreatic ductal adenocarcinoma (PDAC) cases and fatalities are rising worldwide and in the United States, with an approximately 1% annual increase in incidence and correspondingly high mortality. It was ranked as the third leading cause of cancer-related deaths in the United States as of 2020. It is estimated to result in approximately 67,440 new cases and 51,980 deaths in 2025 (1–3).

Despite recent advancements in PDAC treatment and cancer care, patient prognosis remains poor (4–7). Curative-intent radical resection is feasible in less than 15% of patients with localized tumors. For those with borderline resectable, locally advanced, or metastatic disease, chemotherapy is a preferred treatment approach, sometimes used in combination with radiotherapy for locoregional disease (4, 5, 8–10). Over the last 25 years, advances in the treatment of metastatic PDAC have been limited. In 1997, gemcitabine showed a median overall survival (OS) of 5.65 months compared to 5-fluorouracil (11). However, in 2011 the development of new combination chemotherapy regimens has led to an improvement in the prognosis for patients with PDAC. The PRODIGE 4/ACCORD-11 phase III trial assessed the FOLFIRINOX (fluorouracil, leucovorin, irinotecan, and oxaliplatin) regimen versus gemcitabine for metastatic PDAC. Results revealed a median OS of 11.1 months with FOLFIRINOX vs. 6.8 months with gemcitabine (HR 0.57, p<0.001). Regarding toxicity, higher rates of grade 3–4 neutropenia, diarrhea, and neuropathy were reported with FOLFIRINOX. However, patients in the FOLFIRINOX arm had better quality of life at six months (9).

In 2013, the Food and Drug Administration (FDA) approved the combination of gemcitabine plus nanoparticle albumin-bound paclitaxel (nab-paclitaxel) as first-line treatment for metastatic PDAC, followed the findings of the phase III MPACT trial which demonstrated significantly longer OS (8.0 months vs. 6.7 months) than those who received gemcitabine alone (9, 11).

Combinations of chemotherapy, such as FOLFIRINOX and gemcitabine plus nab-paclitaxel (Gem/NabP), have become the first-line standard of care throughout the past few years.

In 2024, the FDA approved fluorouracil, leucovorin, liposomal irinotecan, and oxaliplatin (NALIRIFOX) as a first-line treatment. This approval was based on the NAPOLI 3 trial comparing NALIRIFOX with Gem/NabP. The trial demonstrated improvements in median in both the median OS (11.1 vs. 9.2 months), median progression-free survival (PFS; 7.4 vs. 5.6 months), establishing NALIRIFOX as a new first-line treatment option in this patient population. NALIRIFOX also has distinct safety profile compared with FOLFIRINOX (11).

According to the National Comprehensive Cancer Network (NCCN) Clinical Practice Guidelines in Oncology for metastatic PDAC, recommended first-line treatment options for patients with good performance status, defined as an Eastern Cooperative Oncology Group (ECOG) score of 0-1, include FOLFIRINOX, gemcitabine/nab-paclitaxel, NALIRIFOX (all category 1 recommendations), and mFOLFIRINOX (12). The category 1 designation by the NCCN reflects a high level of evidence, typically derived from randomized controlled trials, and uniform consensus among NCCN panel members that the intervention is appropriate. This classification represents the highest level of endorsement within the NCCN system (12). These Category 1 recommendations apply specifically to metastatic PDAC and should not be extrapolated to locally advanced disease, which has a distinct prognosis and treatment approach.

FOLFIRINOX and NALIRIFOX are both widely used as first-line treatments for metastatic PDAC; however, they have not been directly compared in a head-to-head clinical trial. In the absence of direct comparative evidence, reconstructed individual patient data (rIPD) from published Kaplan-Meier curves can facilitate cross-study comparisons of time-to-event outcomes. By reconstructing patient-level survival data and pooling these data across studies, survival outcomes can be evaluated using regression-based methods while accounting for between-study variability. This approach provides greater resolution of survival patterns over time than comparisons based solely on reported median survival estimates. Other evidence-synthesis approaches, particularly network meta-analysis (NMA), have also been used to address the absence of direct treatment comparisons by integrating direct and indirect evidence through common comparators (13).

A recent meta-analysis by Nichetti et al. (2024) addressed this gap by indirectly comparing NALIRIFOX and FOLFIRINOX through an NMA of phase III trials evaluating both regimens, along with gemcitabine/nab-paclitaxel, in the first-line treatment of metastatic PDAC. Results showed that NALIRIFOX and FOLFIRINOX had comparable PFS (7.4 vs 7.3 months) and OS (11.1 vs 11.7 months), both outperforming Gem/NabP. Notably, NALIRIFOX was associated with lower rates of hematologic toxicity but higher rates of gastrointestinal adverse events (AEs), particularly diarrhea compared with FOLFIRINOX (14).

These findings highlight the value of evidence-synthesis approaches in bridging evidence gaps when direct comparative trials are unavailable. While previous NMAs have provided important indirect comparisons among first-line treatment regimens, rIPD analyses offer a complementary approach by enabling pooled time-to-event analyses using patient-level survival data reconstructed from published Kaplan–Meier curves, thereby providing additional insight into survival patterns across treatment groups beyond comparisons based solely on aggregate summary estimates.

Despite recent therapeutic advances, significant evidence gaps remain. Notably, cost-effectiveness data for available regimens are inconsistently reported and rarely compared directly, limiting the healthcare system’s ability to make value-based decisions. Additionally, safety profiles vary between treatments, but existing studies often lack standardized, quantitative toxicity reporting using criteria such as the Common Terminology Criteria for Adverse Events (CTCAE), limiting the reliability of cross-study comparisons. This gap restricts clinicians, policymakers, and payers from making fully informed, evidence-based treatment choices. Consequently, comprehensive evaluations integrating efficacy and safety outcomes are needed to support personalized, data-driven, and economically sustainable treatment strategies for metastatic PDAC patients.

Recent advances in KRAS-targeted therapies are reshaping the PDAC treatment landscape. Emerging RAS inhibitors, including daraxonrasib, have demonstrated promising activity in metastatic PDAC. These developments further highlight the importance of establishing a robust comparative benchmark for currently available first-line chemotherapy regimens, including NALIRIFOX and FOLFIRINOX (15).

We conducted a reconstructed individual patient data meta-analysis (rIPD-MA) comparing the efficacy and safety, of NALIRIFOX and FOLFIRINOX as first-line treatment for metastatic PDAC. Our goal is to fill the current evidence gap by combining data from available clinical trials and observational studies to provide a comprehensive and integrated comparison of NALIRIFOX and FOLFIRINOX. This assessment will cover multiple critical outcomes, including survival benefits, and toxicity profiles standardized according to CTCAE criteria.

## 2 Methods

### 2.1 Literature search strategy

A systematic literature search was conducted using PubMed, Embase, Scopus, ClinicalTrails.gov, and proceedings and publications from the American Society of Clinical Oncology (ASCO) and European Society of Medical Oncology (ESMO). The search included studies published from January 2011 to May 2025, in the English language. Keywords and Medical Subject Headings (MeSH) used in the search included: PDAC, NALIRIFOX, FOLFIRINOX, OS, progression-free survival (PFS), and AEs.

### 2.2 Eligibility criteria

All prospective clinical trials and high-quality observational studies including case-control and retrospective designs, were eligible for inclusion. Studies included if they enrolled treatment-naïve adult patients with unresectable or metastatic PDAC receiving first-line chemotherapy with either NALIRIFOX or FOLFIRINOX. Studies comparing these regimens to other first-line therapies (such as gemcitabine-based therapies or placebo) were also eligible.

Studies were required to report OS and at least one of the following outcomes: PFS, or AEs based on CTCAE criteria. Conference abstracts published in ASCO or ESMO were considered if they met the criteria.

Studies were excluded if they enrolled patients with previously treated PDAC, resectable disease, or malignancies other than PDAC. Other exclusion criteria included preclinical or non-human studies, case reports, narrative reviews, study protocols without results, non-English publications, and duplicate or overlapping cohorts without novel data.

### 2.3 Study selection

A total of 953 records were identified through database searches and manual reference screening. After removing 466 duplicates, 487 records remained. Of these, 19 full-text articles were assessed for eligibility and met the initial inclusion criteria. A total of 10 were subsequently excluded because they lacked Kaplan-Meier curves suitable for rIPD reconstruction, yielding a final cohort of nine studies included in the analysis.

### 2.4 Data abstraction

Data abstraction was performed independently by two reviewers using a standardized data extraction form, with discrepancies resolved through discussion or consultation with a third reviewer. Extracted data included study characteristics (first author, year of publication, study region, design), patient characteristics (total number of patients, treatment-naïve status, age, performance status, and inclusion/exclusion criteria), and treatment details (regimen type FOLFIRINOX, NALIRIFOX, or mFOLFIRINOX regimens along with dosing details and any reported modifications). Information on comparator arms and key clinical outcomes including median OS, PFS, and grade 3/4 AEs, was also extracted. For studies evaluating an investigational agent in combination with FOLFIRINOX or mFOLFIRINOX, only data from the chemotherapy-alone control arm were extracted and included in the analysis; investigational combination arms were excluded.

### 2.5 Clinical outcomes statistical analysis

All data were collated in Microsoft Excel, and statistical analysis was performed using R software. Individual patient-level time-to-event data for OS and PFS were reconstructed from published Kaplan–Meier curves using the IPDfromKM method (16). OS and PFS were analyzed using the Kaplan-Meier method. The primary endpoint of this rIPD-MA was OS, while the secondary endpoints included PFS and AEs. OS was defined as the time from treatment initiation to last follow-up or death from any cause. PFS was defined as the time interval from treatment initiation to either documented disease progression or death, whichever occurred first. AEs were pooled using random-effects model single-proportion meta-analysis, and subgroup analyses were conducted to compare the incidence of grade 3/4 AEs across treatment groups using chi-square (χ²) tests. Risk of bias in randomized clinical trials was assessed using the Cochrane Risk of Bias (version 2) tool, Sterne JAC et al., while non-randomized interventional studies, were assessed using the Risk Of Bias In Non-randomized Studies - of Interventions version 2 (ROBINS-I V2) tool [Sterne JAC et al. (different study)] was used (17, 18).

### 2.6 Survival comparison

A mixed effects Cox regression model was used to estimate treatment effects on OS and PFS, reported as hazard ratios (HRs) with 95% confidence intervals (CIs).Individual patient’s study data was included as a random variable to account for interstudy differences as demonstrated by previous studies Nichetti et al., Pietrantonio et al., and Raimondi et al. A two-tailed p-value < 0.05 was considered statistically significant (14, 19, 20).

## 3 Results

### 3.1 Study characteristics and selection

Nine studies were included in the main analysis, and 2,733 patients were included in total. 1,138 (42%) patients received FOLFIRINOX, 1,180 (43%) received mFOLFIRINOX, while 415 (15%) patients received NALIRIFOX. Included trial’s characteristics are shown in **Table 1**. The PRISMA flow diagram is shown in **Figure 1**.

**Table 1:** Table of characteristics.

Study name	Year	Country	Study design	Regimen included in analysis	Disease stage/population
NAPOLI-3 (21).	2023	International (18 countries)	Phase III randomized trial	NALIRIFOX	Metastatic PDAC
CISPD3 (24).	2023	China	Phase II randomized trial	mFOLFIRINOX	Metastatic/recurrent PDAC
AVENGER 500 (25).	2024	International (6 countries)	Phase III randomized trial	mFOLFIRINOX	Metastatic PDAC
NEOLAP-AIO-PAK-0113 (27).	2021	Germany	Phase II randomized trial	FOLFIRINOX	Locally advanced PDAC
Wainberg ZA, et al. (26).	2021	USA, Spain, Australia	Phase I/II study	NALIRIFOX	Locally advanced/Metastatic PDAC
Tezuka R, et al. (23).	2022	Japan	Retrospective cohort	mFOLFIRINOX	Unresectable advanced PDAC
Taieb et al. (28).	2023	France, Italy, Germany, Spain, UK	Retrospective observational study	mFOLFIRINOX	Metastatic PDAC
Lee et al. (22).	2020	South Korea	Retrospective cohort	FOLFIRINOX	Metastatic PDAC
Klein-Brill A, et al. (29).	2022	USA	Retrospective comparative effectiveness study	FOLFIRINOX	Metastatic PDAC

**Figure 1:**
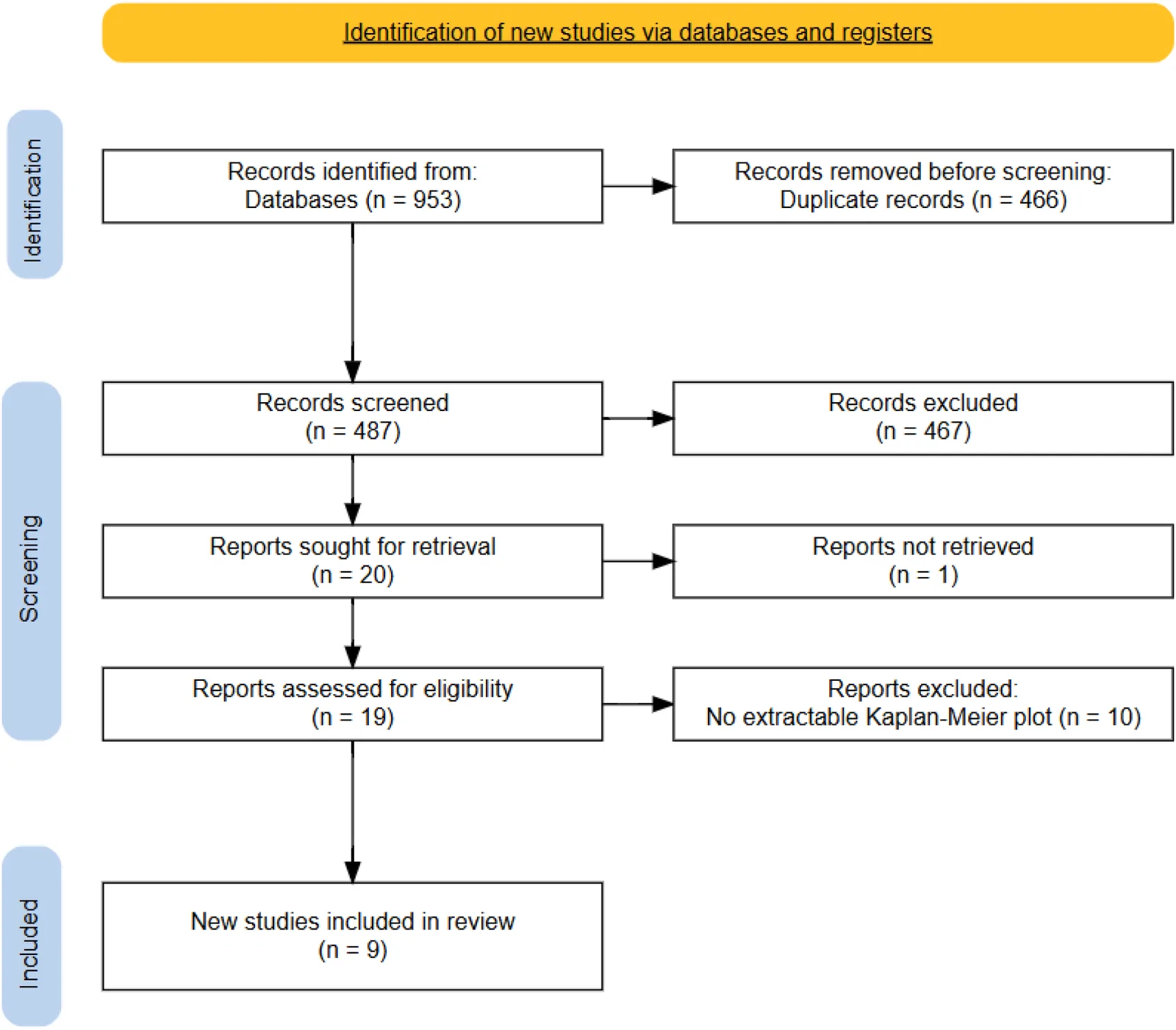
PRISMA flow diagram showing identification and selection of studies: out of 953 records from databases, 466 duplicates were removed, 487 records were screened, 467 excluded, 20 reports sought, 1 not retrieved, 19 assessed for eligibility, 10 excluded due to no extractable Kaplan-Meier plot, and 9 studies included.

Only six studies [Jong-Chan Lee, NAPOLI-3, Ryuichi Tezuka, CISPD3, AVENGER500, Zev A Wainberg] reported PFS, PFS was studied in 1,027 patients, 415 (40%) received NALIRIFOX, 408 (40%) received FOLFIRINOX, and 204 (20%) received mFOLFIRINOX (21–26).

The risk-of-bias assessment identified one study at low risk, three with some concerns, two at moderate risk, and three at serious risk of bias. Detailed assessments are presented in supplementary **eFigure 1**.

### 3.2 Survival outcomes

Overall median OS was 12.38 months (95% CI: 12.06 - 12.85), median OS for the FOLFIRINOX group was 10.98 months, while those received mFOLFIRINOX had a median OS of 14.09 months, and the median OS of the NALIRIFOX group was 11.81 months, **Figure 2A**. Moreover, mFOLFIRINOX and FOLFIRINOX were pooled together with a median OS of 12.59 months, **Figure 2B**.

**Figure 2:**
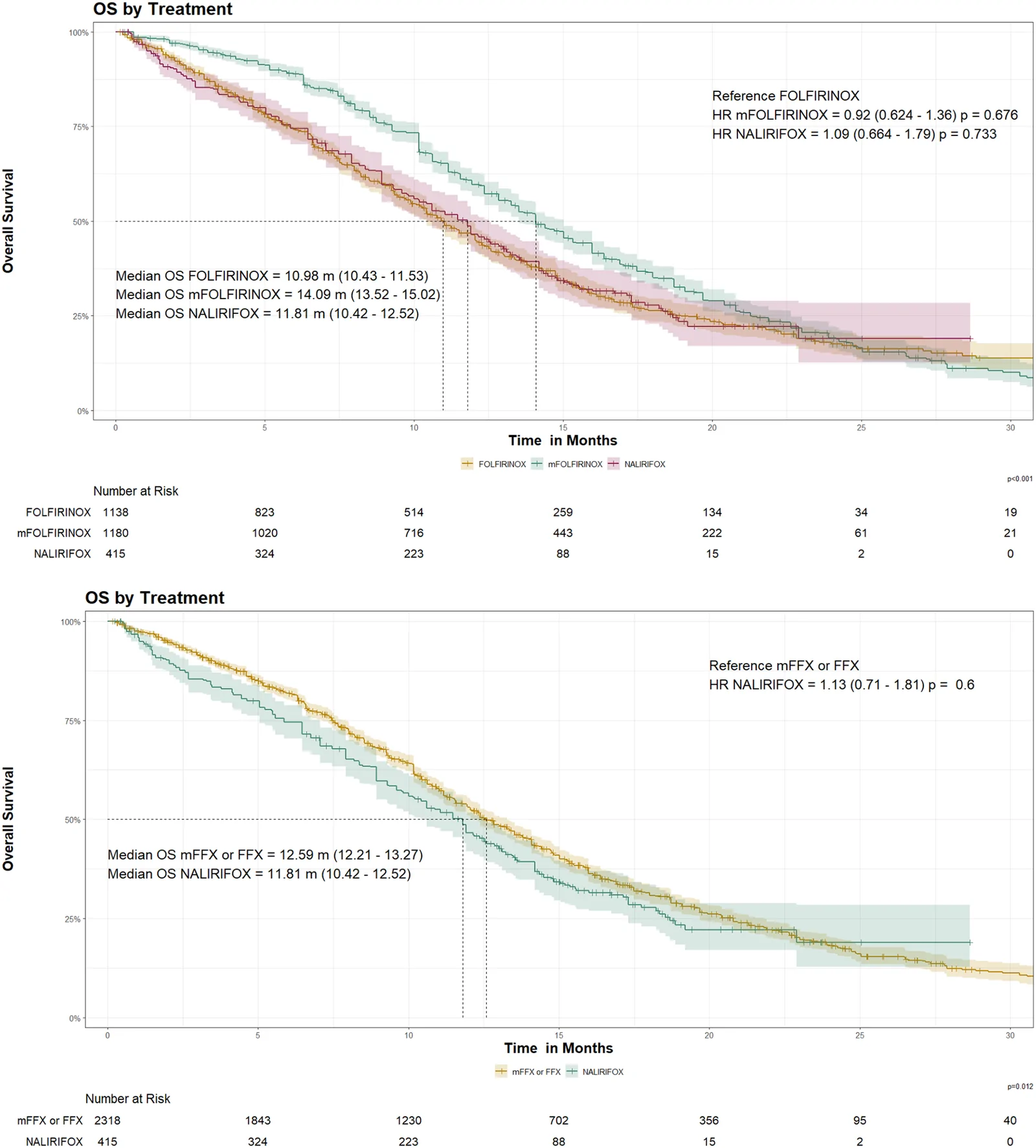
Kaplan Meier curves and hazard ratios comparing **(A)** OS of patients who received NALIRIFOX (NFX) compared to patients who received mFOLFIRINOX (mFFX), FOLFIRINOX (FFX), and **(B)** a combined mFOLFIRINOX (mFFX) or FOLFIRINOX (FFX) arm.

Overall median PFS was 7.66 months (95% CI: 7.30 - 8.21). The mPFS for NALIRIFOX 7.49 months, FOLFIRINOX 8.24 months, and mFOLFIRINOX 6.64 months **Figure 3A**. Additionally, the merged mFOLFIRINOX and FOLFIRINOX group had a mPFS of 7.71 months, **Figure 3B**.

**Figure 3:**
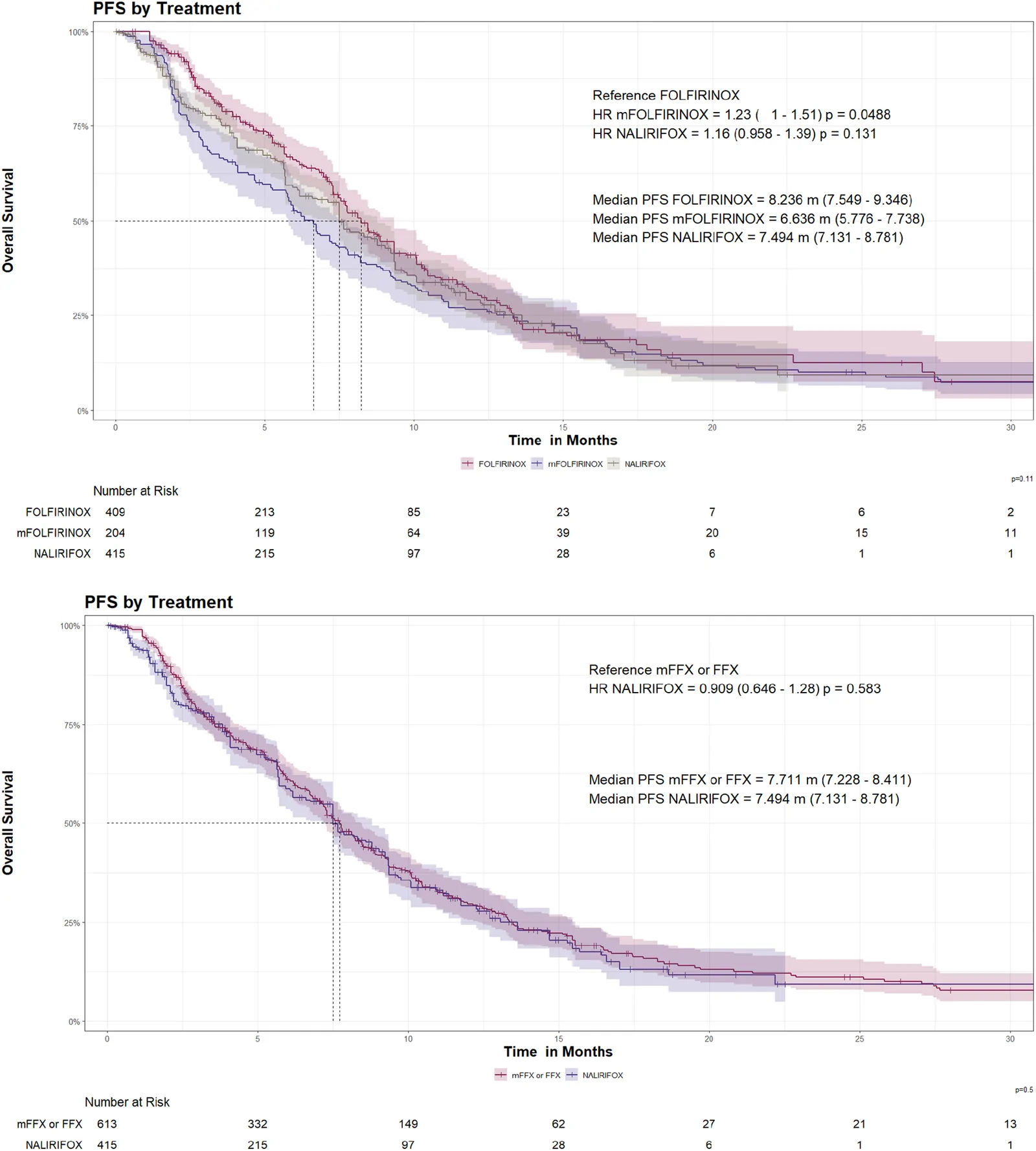
Kaplan Meier curves and hazard ratios comparing **(A)** PFS of patients who received NALIRIFOX (NFX) compared to patients who received mFOLFIRINOX (mFFX), FOLFIRINOX (FFX), and **(B)** a combined mFOLFIRINOX (mFFX) or FOLFIRINOX (FFX) arm.

As shown in **Figures 2**, **3**, there were no significant differences in OS and PFS between NALIRIFOX and the mFOLFIRINOX or FOLFIRINOX combined arm. Additionally, there were no differences in OS for NALIRIFOX and mFOLFIRINOX compared to FOLFIRINOX. There were no significant differences in PFS between NALIRIFOX and FOLFIRINOX, however mFOLFIRINOX had worse PFS compared to FOLFIRINOX (HR 1.23, 95%CI 1.00 - 1.51, p=0.049). Moreover, there were no significant differences in OS and PFS between NALIRIFOX and FOLFIRINOX.

### 3.3 Safety analysis

As shown in **Table 2**, NALIRIFOX had the highest rate of total grade 3/4 AEs, however NALIRIFOX had the lowest rates for decreased white blood cells and neutropenia, vomiting, and febrile neutropenia and it had a low rate for peripheral neuropathy (3.2%). Interestingly, patients treated with NALIRIFOX had the highest rate of grade 3 or 4 diarrhea, as about 20% of patients had it. Patients had comparable rates of anemia and decreased platelets across the 3 different groups. Additionally, patients receiving mFOLFIRINOX reported the lowest rates for fatigue, diarrhea, and peripheral neuropathy.

**Table 2:** Total and specific grade 3/4 adverse events for patients receiving FOLFIRINOX, mFOLFIRINOX, and NALIRIFOX.

AE	FOLFIRINOX	NALIRIFOX	mFOLFIRINOX	p_value
Total	63.1	82.9	52.2	p < 0.001
Fatigue	15.1	15.1	3.7	0.112
Peripheral Neuropathy	8.2	3.2	0	0.0121
WBC Decrease	43.7	18.9	44.4	0.0177
Anemia	6.6	10.4	13	0.33
Platelets Decrease	4.8	10.5	11.1	0.278
Diarrhea	9.7	19.7	3.7	0.006
Vomiting	24.1	7.2	11.1	p < 0.001
Febrile Neutropenia	13.4	4.7	NA	0.0886

## 4 Discussion

This comprehensive study represents a deep comparison of NALIRIFOX and FOLFIRINOX which are both standard first-line treatment options for metastatic PDAC, integrating survival outcomes, and standardized toxicity data per CTCAE criteria. Our results suggest that when FOLFIRINOX and mFOLFIRINOX are pooled, the combined FOLFIRINOX group achieves a numerically higher median OS (12.59 months; 95% CI: 12.21–13.27) compared with NALIRIFOX (11.81 months; 95% CI: 10.42–12.52), a difference that did not reach statistical significance (HR 1.13, 95% CI: 0,71 –1.81; p = 0.6). However, upon disaggregation, this survival advantage is largely driven by the mFOLFIRINOX subgroup (median OS: 14.09 months, 95% CI: 13.52–15.02), while full-dose FOLFIRINOX (mOS: 10.43 months) was associated with a numerically inferior OS compared with NALIRIFOX.

### 4.1 Survival outcomes

Our survival analyses are consistent with the currently available body of evidence yet add important resolution by disaggregating FOLFIRINOX variants. The findings from the landmark PRODIGE 4/ACCORD-11 phase III trial established FOLFIRINOX (OS: 11.1 months; HR vs. gemcitabine, 0.57; p < 0.001) as a cornerstone of first-line therapy for fit patients with metastatic PDAC (30).The NAPOLI 3 phase III trial subsequently demonstrated that NALIRIFOX achieved a median OS of 11.1 months and PFS of 7.4 months, both superior to Gem/NabP (OS 9.2 months; PFS 5.6 months) (21). Consistent with the NMA by Nichetti et al., which found statistically comparable OS and PFS between NALIRIFOX (OS 11.1 months; PFS 7.4 months) and FOLFIRINOX (OS 11.7 months; PFS 7.3 months) through indirect comparison of phase III trials, our analysis further confirms the absence of a meaningful OS difference when comparing NALIRIFOX with full-dose FOLFIRINOX alone (HR = 1.09, 95% CI: 0.66 –1.79; p = 0.73) (14).

The superior OS observed with mFOLFIRINOX in our cohort warrants careful interpretation as the higher OS in this subgroup may reflect the characteristics of populations enrolled in retrospective studies that selectively report outcomes in patients who tolerate dose-modified regimens. These findings may be supported by real-world registry data suggesting that mFOLFIRINOX achieves favorable outcomes, partly attributable to patient selection and oncologist experience with proactive dose management, as the systematic review by Cockrum et al. demonstrates (11). Therefore, the observed OS advantage with mFOLFIRINOX should be interpreted cautiously and should not be considered evidence of superiority over NALIRIFOX or FOLFIRINOX.

The Mastrantoni et al. Bayesian NMA, published in The Lancet Oncology, similarly identified NALIRIFOX among the preferred regimens alongside FOLFIRINOX, both offering superior PFS and OS over doublet therapies (1).

Regarding PFS, FOLFIRINOX demonstrated a numerically higher median PFS (8.24 months; 95% CI: 7.55–9.35) compared with NALIRIFOX (7.49 months; 95% CI: 7.13–8.78), whereas mFOLFIRINOX exhibited the lowest median PFS (6.64 months; 95% CI: 5.78–7.74). The pooled FOLFIRINOX/mFOLFIRINOX group achieved a median PFS of 7.71 months, numerically comparable to NALIRIFOX. The overall median PFS across all groups was 7.66 months (95% CI: 7.30–8.21). These data suggest that the PFS advantage of full-dose FOLFIRINOX is offset in the mFOLFIRINOX cohort, which may reflect dose attenuation and the selection of patients with more advanced or comorbid disease profiles in real-world settings.

### 4.2 Safety and toxicity

The toxicity profiles of NALIRIFOX and FOLFIRINOX represent a clinically relevant dimension of their comparison, as treatment tolerability directly influences dose intensity, patient quality of life, and healthcare resource utilization. Our analysis demonstrates distinct CTCAE-standardized toxicity signatures for the two regimens, aligning with patterns reported in prior work (14).

FOLFIRINOX is characterized by a high burden of hematologic toxicity, most notably grade 3–4 neutropenia (reported in up to 45–53% of patients in clinical trial data) and febrile neutropenia, which frequently necessitates treatment delays, dose reductions, and granulocyte colony-stimulating factor (G-CSF) support (30, 31). NALIRIFOX, by contrast, incorporates liposomal irinotecan at a lower dose, alongside reduced-dose oxaliplatin. This formulation confers a measurably lower incidence of high-grade hematologic toxicity; with grade 3/4 thrombocytopenia reported at 1.6% with NALIRIFOX versus 11.8% with FOLFIRINOX in the meta-analysis by Nichetti et al. (14). Conversely, NALIRIFOX is associated with substantially higher rates of grade 3–4 diarrhea (approximately 20.3% in NAPOLI 3), which may impair quality of life, precipitate hypokalemia (grade 3–4 incidence of 15.1%), and contribute to electrolyte disturbances requiring hospitalization (21).

The real-world safety data for NALIRIFOX is reassuring. A first multicenter Western real-world cohort published in ESMO Open reported that only 22.5% of patients experienced grade 3–4 AEs, lower than the rates observed in the controlled NAPOLI 3 trial environment, suggesting that proactive toxicity management in routine clinical practice can attenuate treatment-emergent severe events (32).The neurological toxicity burden, principally cumulative peripheral neuropathy attributable to oxaliplatin, represents a shared concern for both regimens, though NALIRIFOX’s reduced oxaliplatin dosing may delay or attenuate the onset of clinically significant neuropathy compared with full-dose FOLFIRINOX.

The standardization of toxicity reporting using CTCAE criteria across studies in our paper is a methodological strength that enables more reliable cross-study comparison. Nevertheless, variability in the completeness of AE reporting across included studies, including missing data for specific toxicities, remained a limitation. Differences in AE collection and reporting between prospective trials and retrospective cohorts may also have influenced the observed toxicity rates; therefore, cross-study safety comparisons should be interpreted cautiously.

### 4.3 Clinical implications and treatment selection

The totality of evidence from this rIPD-MA supports the view that NALIRIFOX and FOLFIRINOX (both as full-dose and in modified form) represent viable first-line options for fit patients with metastatic PDAC, with OS outcomes that are numerically similar and not statistically different when full-dose FOLFIRINOX is used as the comparator. The apparent OS advantage of the pooled FOLFIRINOX and mFOLFIRINOX group is principally attributable to the mFOLFIRINOX subgroup, which likely reflects real-world patient selection effects and dose-modification practices in high-volume expert centers.

From a clinical decision-making standpoint, the distinct toxicity profiles of these regimens should drive individualized treatment selection. NALIRIFOX may be preferentially suited to patients in whom hematologic toxicity is of concern, such as those with borderline bone marrow reserve, prior pelvic radiation, or limited access to G-CSF support, while its higher rate of grade 3–4 diarrhea and hypokalemia necessitate proactive bowel management and electrolyte monitoring.

Quality of life and cost-effectiveness are also important considerations when selecting first-line therapy. Differences in toxicity profiles, supportive care requirements, treatment administration, and healthcare resource utilization may influence both patient quality of life and overall treatment costs. However, comparative quality-of-life and cost-effectiveness data for NALIRIFOX and FOLFIRINOX remain limited, highlighting the need for prospective studies incorporating patient-reported and economic outcomes.

An additional consideration in treatment selection is the pharmacogenomic variability in irinotecan metabolism across racial populations. The UGT1A1 enzyme, responsible for irinotecan detoxification, exhibits markedly different allele frequency distributions between Asian and Western populations (33). Specifically, the UGT1A1 *6 variant, which impairs irinotecan glucuronidation and increases toxicity risk, is substantially more prevalent in East Asian populations, while the UGT1A1 *28 variant predominates in Western cohorts (22, 23). This distinction is clinically relevant to the current analysis, as several included studies enrolled predominantly Asian populations and the observed rates of grade 3/4 diarrhea and hematologic toxicity with both NALIRIFOX and mFOLFIRINOX may not be fully generalizable across racial groups (22, 23). Clinicians treating Asian patients with either irinotecan-containing regimen should consider UGT1A1 genotyping to inform starting dose selection and anticipate differential toxicity burdens.

Furthermore, the presence of homologous recombination repair mutations (HRRm) warrants explicit consideration when interpreting the OS outcomes reported in this analysis. The pooled OS figures represent composite signals across molecularly heterogeneous populations, and HRRm prevalence differences across included study cohorts may partially account for the OS variation observed, particularly within the mFOLFIRINOX subgroup. Real-world data from Reichinger et al. demonstrate that HRRm-positive patients treated with NALIRIFOX achieved a median OS of 16.24 months compared with 11.11 months in HRRm-wildtype patients a difference of substantial clinical magnitude that would meaningfully shift the interpretation of regimen-level OS estimates if HRRm enrichment varied systematically across cohorts. Prospective biomarker-stratified trials are therefore essential to disentangle the independent contributions of regimen choice and tumor molecular profile to survival outcomes in this setting.

Although this rIPD meta-analysis was not designed to evaluate molecular resistance, both NALIRIFOX and FOLFIRINOX may share resistance mechanisms because they contain 5-fluorouracil, oxaliplatin, and an irinotecan-based component. Potential irinotecan-related mechanisms include alterations in TOP1, drug transport, and DNA-damage response pathways, while UGT1A1 variation primarily affects SN-38 exposure and toxicity rather than established acquired resistance. Resistance to 5-fluorouracil may involve TYMS and altered fluoropyrimidine metabolism, whereas oxaliplatin resistance may involve DNA-repair pathways including ERCC1, BRCA1/2, PALB2, and RAD51. Emerging FOLFIRINOX-resistant PDAC models also implicate metabolic and transport pathways, including NET1 and SLC2A1. These mechanisms should be considered biologically plausible rather than validated NALIRIFOX-specific biomarkers. Prospective studies with serial tumor sequencing and circulating tumor DNA are needed to define regimen-specific acquired resistance.

Emerging KRAS-targeted therapies may further influence first-line treatment strategies in PDAC. As these agents are evaluated in combination with chemotherapy, the present findings provide a benchmark for the efficacy and safety of established chemotherapy regimens (15). The findings of this rIPD-MA thus provide a timely benchmark of chemotherapy backbone performance against which future KRAS inhibitor-based combination regimens can be contextualized.

### 4.4 Strengths and limitations

A primary strength of our study is the utilization of an IP fromKM method to reconstruct individual patient-level survival data from published Kaplan–Meier curves.This methodological approach allows for a more granular assessment of survival over time than standard aggregate data meta-analyses, providing a more precise estimation of the targeted endpoints. Furthermore, the inclusion of a large number of patients (over 2,000) provides the statistical power necessary to discern subtle but clinically significant differences in safety and efficacy.

This rIPD-MA is subject to several important limitations that warrant acknowledgment. First, the absence of a direct head-to-head randomized controlled trial comparing NALIRIFOX and FOLFIRINOX is the foundational limitation of all indirect comparative analyses in this field; while NMA preserves the benefits of randomization within individual trials, residual confounding from indirect comparisons cannot be fully eliminated. Second, the inclusion of both clinical trials and retrospective observational studies in the analysis introduces potential selection bias and heterogeneity in patient populations, with observational cohorts potentially enrolling patients with better performance status or at higher-volume centers, which may particularly affect the mFOLFIRINOX subgroup’s favorable outcomes. Third, adverse event data in retrospective studies were frequently incompletely reported or inconsistently graded, limiting the comprehensiveness of the safety synthesis. Additionally, outcome data on quality of life, patient-reported outcomes, and downstream treatment sequencing were insufficiently reported across studies to enable formal pooling. Finally, the included studies generally did not provide serial tumor genomic, transcriptomic, or circulating tumor DNA profiling before and after treatment. Therefore, our analysis cannot directly identify acquired resistance mechanisms or determine whether specific molecular alterations differentially emerge following NALIRIFOX versus FOLFIRINOX exposure. The resistance pathways discussed in this manuscript are therefore mechanistic hypotheses derived from the pharmacology of the individual regimen components and the broader PDAC chemoresistance literature rather than findings directly generated by the present rIPD analysis.

## 5 Conclusion

This rIPD-MA comprehensively compares the efficacy and safety of NALIRIFOX versus FOLFIRINOX and mFOLFIRINOX as first-line therapy for mPDAC. No statistically significant differences in OS or PFS were observed between NALIRIFOX and FOLFIRINOX, however, these findings do not establish therapeutic equivalence or non-inferiority. NALIRIFOX is associated with lower hematological toxicity but with higher rates of severe diarrhea, while FOLFIRINOX shows the opposite pattern. Both regimens are valid first-line options for fit patients. Treatment selection should be individualized based on expected toxicities, comorbidities, performance status, and institutional resources. Prospective head-to-head trials incorporating serial tumor sequencing and circulating tumor DNA analyses are needed to determine whether NALIRIFOX and FOLFIRINOX exert distinct molecular selective pressures and to identify biomarkers of acquired resistance.

## Data Availability

The original contributions presented in the study are included in the article/**Supplementary Material**. Further inquiries can be directed to the corresponding author.

## Supplementary material

Supplementary Figure 1 **(A)** Risk of bias 2 assessment of trials included in this study. **(B)** Risk of bias assessment for interventional randomized studies included in this study.
