# Supplementary material for: NALIRIFOX versus FOLFIRINOX: reconstructed individual patient data meta-analysis of efficacy and safety in first-line treatment of unresectable or metastatic pancreatic ductal adenocarcinoma
Source: Front Oncol. 2026 Sep 18;16:1902839. doi: 10.3389/fonc.2026.1902839 (PMC13630592; doi:10.3389/fonc.2026.1902839)
Supplement: Supplementary file 1 [file SupplementaryFile1.docx]

**NALIRIFOX Versus FOLFIRINOX:** **Reconstructed Individual Patient Data Meta-Analysis of Efficacy and Safety in First-Line Treatment of Unresectable or Metastatic Pancreatic Ductal Adenocarcinoma**

**Abdullah Esmail ^1, 2^,** **Jian Guan^1, 2^, Zaid Alabed^3^, Yazan Hamdaneh ^3^ Ebtesam Al-Najjar^3^ Saifudeen Abdelrahim^1^, Nour Mustafa ^3^, Bayan Khasawneh^1^ and Maen Abdelrahim ^1, 2, 3*^**

1	Section of GI Oncology, Department of Medicine, Houston Methodist Cancer Center, Houston, TX 77030, USA

2	Department of Medicine, Weill Cornell Medical College, NY, NY, 10065 USA

3	Faculty of Medicine, the University of Jordan, Amman 11942, Jordan

*	Correspondence: mabdelrahim@houstonmethodist.org

eFigure 1A: Risk of bias 2 assessment of trials included in this study

eFigure 1B: Risk of bias assessment for interventional randomized studies included in this study
